# Research on Transfer Learning of Vision-based Gesture Recognition

**DOI:** 10.1007/s11633-020-1273-9

**Published:** 2021-03-08

**Authors:** Bi-Xiao Wu, Chen-Guang Yang, Jun-Pei Zhong

**Affiliations:** 1 College of Automation Science and Engineering, South China University of Technology, Guangzhou, 510640 China; 2 Shien-Ming Wu School of Intelligent Engineering, South China University of Technology, Guangzhou, 511442 China; 3 Foshan Newthinking Intelligent Technology Company Ltd., Foshan, 528231 China

**Keywords:** Transfer learning, gesture recognition, red-green-blue (RGB) Camera, Leap Motion, joint distribution adaptation (JDA)

## Abstract

Gesture recognition has been widely used for human-robot interaction. At present, a problem in gesture recognition is that the researchers did not use the learned knowledge in existing domains to discover and recognize gestures in new domains. For each new domain, it is required to collect and annotate a large amount of data, and the training of the algorithm does not benefit from prior knowledge, leading to redundant calculation workload and excessive time investment. To address this problem, the paper proposes a method that could transfer gesture data in different domains. We use a red-green-blue (RGB) Camera to collect images of the gestures, and use Leap Motion to collect the coordinates of 21 joint points of the human hand. Then, we extract a set of novel feature descriptors from two different distributions of data for the study of transfer learning. This paper compares the effects of three classification algorithms, i.e., support vector machine (SVM), broad learning system (BLS) and deep learning (DL). We also compare learning performances with and without using the joint distribution adaptation (JDA) algorithm. The experimental results show that the proposed method could effectively solve the transfer problem between RGB Camera and Leap Motion. In addition, we found that when using DL to classify the data, excessive training on the source domain may reduce the accuracy of recognition in the target domain.

### 1 Introduction

Recently, human-robot interaction has been developed rapidly. Gesture could be an important way for human-robot interaction since it is able to give accurate and intuitive instruction to the robots, and it has been widely studied for decades[1]. Gesture recognition could enable effective and efficient interactions between human workers and robots. There are many kinds of devices for vision-based gesture recognition. For example, the camera is the main sensor used in the field of gesture recognition. Previously, most of the researchers used red-green-blue (RGB) images for gesture recognition[2]. With the development of technology, some new devices have sprung up, such as leap motion, Kinect, etc. Leap motion is an interactive hardware device based on infrared radiation (IR) sensors, and it could precisely capture and extract the positions and angles of finger joints. Specifically, Leap Motion is designed to detect and track human hand gestures, so the error of tracking is about 200 µm about the 3D coordinate of fingertips[3].

However, the data from different domains may be distributed differently. Therefore, classifiers trained from one domain are likely to have a poor performance in the other domains. And for each domain, it is too expensive to collect a mass of examples manually and build a separate classifier. Therefore, how to make better use of the trained model in the source domain and reduce the learning cost in the target domain has become an urgent problem to be solved.

In recent years, transfer learning has arisen wide interest in researchers. Transfer learning refers to the application of existing knowledge to other related domains. Researchers have studied transfer learning in different methods, e.g., board learning system (BLS)[4, 5], neural network (NN)[6], Bayesian model[7] and some other methods. Although transfer learning has received a lot of attention in [8], there are very few cases in the application of gesture recognition. The goal of this paper is to propose a method in the field of gesture recognition, which enables a model trained in the source domain to be used in the target domain directly. Therefore, the time for collecting data is reduced and the time for annotating data could be minimized or eliminated[9].

Research Article
Manuscript received November 20, 2020; accepted December 23, 2020; published online March 8, 2021
Recommended by Associate Editor Hui Yu
Colored figures are available in the online version at https://link.springer.com/journal/11633
© The author(s) 2021

At present, transfer learning has been effectively used in text classification[10, 11], sentiment classification[12−14], image classification[15−21] and other fields. It could be divided into feature representation transfer learning, instance transfer learning, parameter transfer learning and relationship knowledge transfer learning[8]. Feature representation transfer learning refers to transfer through feature transformation to decrease the difference between the source domain and the target domain[22−24]; or to convert the data of the source and target domains into a unified feature space[25−27], and then use the classification algorithm for identification. Feature representation transfer learning is one of the most popular research methods in the field of transfer learning. The paper uses this method to convert the original data of the RGB Camera and Leap Motion into a unified feature space, and then use the classification algorithm for recognition.

In the process of gesture recognition, it is generally necessary to assume: 1) the same feature space, it means that the training and test data need to use the same set of sensors; 2) the same overall distribution, i.e., experimenters′ preferences or habits on training and test data are similar; 3) the same label space, i.e., the same label set in the training and test data[25]. Using conventional unsupervised data mining methods for gesture recognition, the long data collection cycle becomes a practical problem. If a supervised method is used, it will put a great burden on users, and they must annotate enough data to train the algorithm. It is a time-consuming task to label the original sensor data manually and requires experts to spend a lot of time annotating the sensor data. In addition, learning the model of each device independently and neglecting the learned knowledge in other domains will result in redundant calculation workload, excessive time cost, and loss of useful knowledge. Consequently, it is very profitable to develop models in a new field by using the learned information. Using transferable knowledge could decrease the collection of data, reduce the need for data annotation, and increase the learning speed[9]. There is very little work to transfer knowledge between two or more sensor models. Kurz et al.[28] and Roggen et al.[29] used teacher/learner models to handle the transfer problem of action recognition. Hu and Yang[24] introduced a transfer learning method to effectively transfer knowledge between models, but the greater knowledge transfer between different domains remains to be explored. Marin et al.[30] proposed how to jointly exploit the Camera and Leap Motion for accurate gesture recognition. However, it still needs to collect a large number of data from various devices and does not use the model learned from a certain device. The focus of this paper is to effectively solve the transfer problem between the RGB Camera and Leap Motion, thereby improving the learning efficiency of cross-device transfer.

This paper presents a method to apply the learned model in one device to another. The RGB Camera and Leap Motion were used to collect gesture data from several human users to verify the presented method. The main contributions are as follows:

1) A transfer learning framework of gesture recognition across different devices is proposed. Here, these devices have different data distributions, but all of them have the same output labels.

2) In the transfer of gesture recognition by the RGB Camera and Leap Motion, we extract several different features, and from the experimental results, the average accuracy of the new coordinate features is the highest.

3) When using the back propagation neural network (BP NN) algorithm for classification, we found that in some cases, the epoch of training has some unusual effects on the transfer results. Too many training times may lead to model overfitting in the source domain, and reduce the generalization ability in the target domain.

Fig. 1 shows a general overview of our approach. The

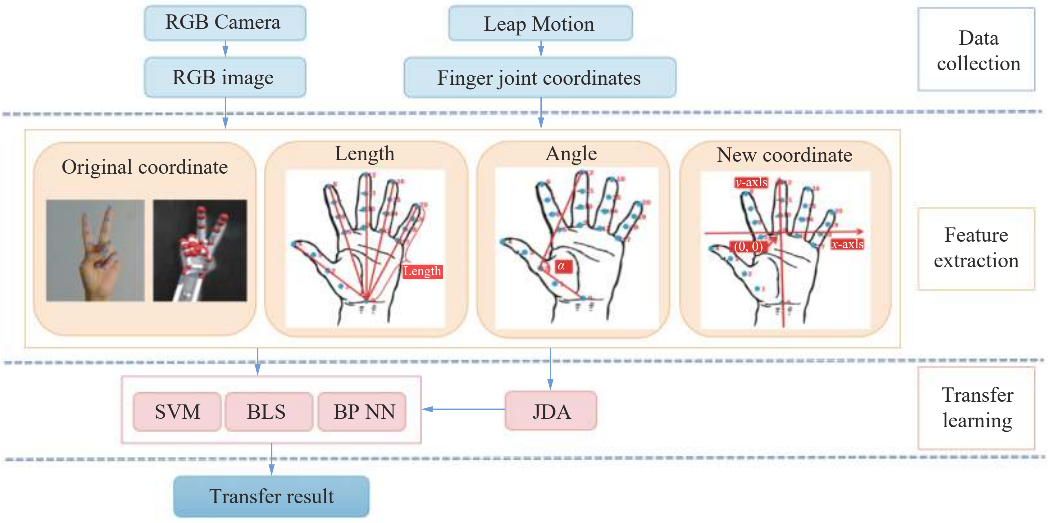

Fig. 1 Pipeline of the proposed approach structure of this paper is organized as follows. In Section 2, the preliminaries of transfer learning are reviewed. In Section 3, the data collection and feature extraction are described. Then, we introduce the experiment in Section 4. We further discuss the problems found in the experiment in Section 5, and Section 6 concludes our work.

### 2 Preliminaries

#### 2.1 Joint distribution adaptation (JDA)[31]

The difference between the source domain and the target domain is approximated by the distance between $P(x_s)$ and $P(x_t)$, and the distance between $P(y_s|x_s)$ and $P(y_t|x_t)$, as shown in (1). The JDA algorithm realizes transfer by reducing the distance of marginal distribution and conditional distribution in different domains. In this paper, we use the JDA algorithm to reduce the distance between the RGB Camera and Leap Motion. Just to be clear, the related notations and descriptions are shown in Table 1.

$$d(D_s, D_t) \approx ||P(x_s) - P(x_t)|| + ||P(y_s|x_s) - P(y_t|x_t)||. \quad (1)$$

##### 2.1.1 Feature transformation

Dimensionality reduction could be used to transfer the data. For clarity, principal component analysis (PCA) is used to reconstruct the data. The goal of PCA is to find a transformation matrix $\boldsymbol{A}$ to maximize the embedded data variance, which is shown in (2).

Table 1 Notations and descriptions

| Notation | Description |
|---|---|
| $D_s, D_t$ | Source/Target domain |
| $N_s, N_t$ | Source/Target dimension |
| $\boldsymbol{X}$ | Input data matrix |
| $\boldsymbol{A}$ | Adaptation matrix |
| $\boldsymbol{Z}$ | Embedding matrix |
| $\boldsymbol{H}$ | Lefting matrix |
| $\boldsymbol{M}_c$ | MMD matrices, $c \in [0, \cdots, C]$ |
| $\boldsymbol{\Phi}$ | $k$ largest eigenvalues |
| $p$ | Mapping feature node |
| $n$ | Number of mapping features |
| $q$ | Number of enhanced feature nodes |
| $(x_{ci}, y_{ci})$ | The hand joint point coordinates obtained by the RGB Camera, $c_i \in [0, 1, \cdots, 20]$ |
| $(x_{li}, y_{li})$ | The hand joint point coordinates obtained by the Leap Motion, $l_i \in [0, 1, \cdots, 20]$ |
| $length$ | The length feature |
| $\alpha$ | The angel feature |
| $C$ | SVM penalty coefficient |

$$\max_{\boldsymbol{A}^{\rm T}\boldsymbol{A}=\boldsymbol{I}} {\rm tr}(\boldsymbol{A}^{\rm T}\boldsymbol{XHX}^{\rm T}\boldsymbol{A}). \quad (2)$$

Eigen decomposition $\boldsymbol{XHX}^{\rm T}\boldsymbol{A} = \boldsymbol{A\Phi}$ can deal with this optimization problem effectively.

##### 2.1.2 Marginal distribution adaptation

Although PCA could extract $k$-dimensional features from the data, the distribution of different domains is still very large. It needs to reduce the difference of marginal distributions firstly, in other words, the distance between $P(\boldsymbol{A}^t x_s)$ and $P(\boldsymbol{A}^t x_t)$ should be as close as possible. The maximum mean discrepancy (MMD)[32] is used to compute the distance between the source domain and the target domain.

$$\left|\left|\frac{1}{n_s}\sum_{i=1}^{n_s}\boldsymbol{A}^{\rm T}x_i - \frac{1}{n_t}\sum_{j=n_s+1}^{n_s+n_t}\boldsymbol{A}^{\rm T}x_j\right|\right|^2 = {\rm tr}(\boldsymbol{A}^{\rm T}\boldsymbol{XM}_0\boldsymbol{X}^{\rm T}\boldsymbol{A}) \quad (3)$$

where $\boldsymbol{M}_0$ is the MMD matrix and is computed as follows:

$$(\boldsymbol{M}_0)_{ij} = \begin{cases} \dfrac{1}{n_s n_s}, & \text{if } x_i, x_j \in D_s \\ \dfrac{1}{n_t n_t}, & \text{if } x_i, x_j \in D_t \\ \dfrac{-1}{n_s n_t}, & \text{otherwise.} \end{cases} \quad (4)$$

##### 2.1.3 Conditional distribution adaptation

Then, it needs to reduce the difference of the conditional distribution, i.e., the distance between $P(y_s|\boldsymbol{A}^{\rm T}x_s)$ and $P(y_t|\boldsymbol{A}^{\rm T}x_t)$ should be reduced. A modified MMD is used to measure the distance between the $P(y_s|\boldsymbol{A}^{\rm T}x_s)$ and $P(y_t|\boldsymbol{A}^{\rm T}x_t)$.

$$\left|\left|\frac{1}{n_s^{(c)}}\sum_{x_i\in D_s^{(c)}}\boldsymbol{A}^{\rm T}x_i - \frac{1}{n_t^{(c)}}\sum_{x_j\in D_t^{(c)}}\boldsymbol{A}^{\rm T}x_j\right|\right|^2 = tr(\boldsymbol{A}^{\rm T}\boldsymbol{XM}_c\boldsymbol{X}^{\rm T}\boldsymbol{A}) \quad (5)$$

where $\boldsymbol{M}_c$ is computed as follows:

$$(\boldsymbol{M}_c)_{ij} = \begin{cases} \dfrac{1}{n_s^{(c)}{n_s}^{(c)}}, & \text{if } x_i,\ x_j \in D_s^{(c)} \\ \dfrac{1}{n_t^{(c)}{n_t}^{(c)}}, & \text{if } x_i,\ x_j \in D_t^{(c)} \\ \dfrac{-1}{n_s^{(c)}n_t^{(c)}}, & \text{if } \begin{cases} x_i \in D_s^{(c)},\ x_j \in D_t^{(c)} \\ x_j \in D_s^{(c)},\ x_i \in D_t^{(c)} \end{cases} \\ 0, & \text{otherwise.} \end{cases} \quad (6)$$

##### 2.1.4 Optimization problem

In JDA, the distance of the marginal distributions and conditional distributions is minimized at the same time, which makes the transfer learning more robust. Thus, by combining the above two distances, a total optimization goal could be obtained:

$$\min \sum_{c=0}^{C} \mathrm{tr}(\boldsymbol{A}^{\mathrm{T}}\boldsymbol{X}\boldsymbol{M}_c\boldsymbol{X}^{\mathrm{T}}\boldsymbol{A}) + \lambda||\boldsymbol{A}||_F^2. \tag{7}$$

Since the variance of the data is maintained before and after the transformation, another constraint is obtained as

$$\max \boldsymbol{A}^{\mathrm{T}}\boldsymbol{X}\boldsymbol{H}\boldsymbol{X}^{\mathrm{T}}\boldsymbol{A}. \tag{8}$$

Therefore, by combining the above constraints, the optimization goal is transformed into

$$\min \frac{\sum_{c=0}^{C} \mathrm{tr}(\boldsymbol{A}^{\mathrm{T}}\boldsymbol{X}\boldsymbol{M}_c\boldsymbol{X}^{\mathrm{T}}\boldsymbol{A}) + \lambda||\boldsymbol{A}||_F^2}{\boldsymbol{A}^{\mathrm{T}}\boldsymbol{X}\boldsymbol{H}\boldsymbol{X}^{\mathrm{T}}\boldsymbol{A}}. \tag{9}$$

Using the Rayleigh quotient, (9) could be translated as follows:

$$\begin{aligned}&\min \sum_{c=0}^{C} \mathrm{tr}(\boldsymbol{A}^{\mathrm{T}}\boldsymbol{X}\boldsymbol{M}_c\boldsymbol{X}^{\mathrm{T}}\boldsymbol{A}) + \lambda||\boldsymbol{A}||_F^2\\ &\text{s.t. } \boldsymbol{A}^{\mathrm{T}}\boldsymbol{X}\boldsymbol{H}\boldsymbol{X}^{\mathrm{T}}\boldsymbol{A} = \boldsymbol{I}.\end{aligned} \tag{10}$$

According to the Lagrange method, the formula turns out to be as follows:

$$\left(\boldsymbol{X}\sum_{c=0}^{C}\boldsymbol{M}_c\boldsymbol{X}^{\mathrm{T}} + \lambda\boldsymbol{I}\right)\boldsymbol{A} = \boldsymbol{X}\boldsymbol{H}\boldsymbol{X}^{\mathrm{T}}\boldsymbol{A}\boldsymbol{\varPhi}. \tag{11}$$

Thus, we could use the eigs function in Matlab to solve the transformation matrix $\boldsymbol{A}$ easily.

### 3 Feature extraction and selection

We use the RGB Camera and Leap Motion to collect 10 static gestures of multiple experimenters (Figs. 2 and 3), and about 800 sets of data. In order to find the most suitable features for transfer between the two devices, we extract various features from the original data obtained for experimental comparison. We introduce each feature in the following sections.

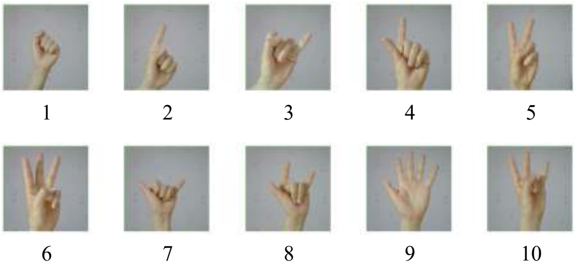

Fig. 2 Ten gestures captured by the RGB Camera

[1]https://ai.baidu.com/tech/body/hand

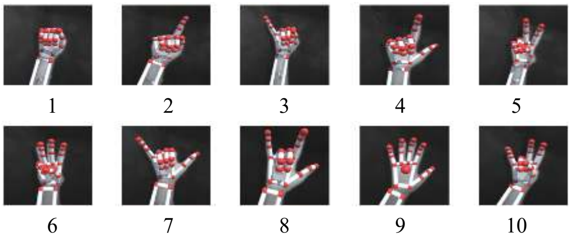

Fig. 3 Ten gestures captured by the Leap Motion

#### 3.1 Feature 1: The coordinates

Thanks to the existing hand key point detection technology, it is easy to extract the coordinates of 21 joint points of the hand from the gesture images taken by the RGB Camera[1], as shown in Fig. 4. We use $(x_{ci}, y_{ci})$, $i = 0, 1, 2, \cdots, 20$ to represent the hand joint point coordinates obtained by the RGB Camera. The upper left corner of the image is the origin of the coordinate system, and the positive direction of the $x$-axis and $y$-axis are shown in Fig. 4. Leap Motion could directly collect the three-dimensional coordinate positions of the 21 joint points of the hand. Fig. 5 is a coordinate system with the center of the Leap Motion device as the origin of the coordinates. In the paper, $(x_{li}, y_{li})$, $i = 0, 1, 2, \cdots, 20$ represents the hand joint point coordinates obtained by Leap Motion, and the depth information is not used in this work.

The joint point coordinates extracted from the RGB Camera and Leap Motion corresponding to the position of the hand are shown in Fig. 6. It could be seen that the coordinates obtained by the two devices correspond to the same joint points. However, the coordinate systems of the two devices are different, so their distributions are different.

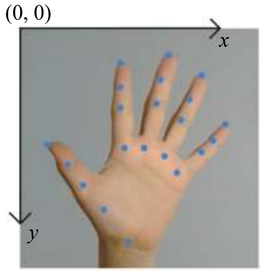

Fig. 4 Hand joints coordinate obtained from the picture

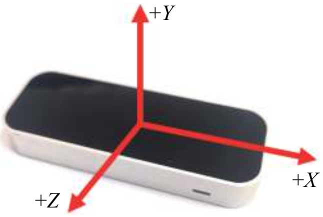

Fig. 5 Leap Motion coordinate system

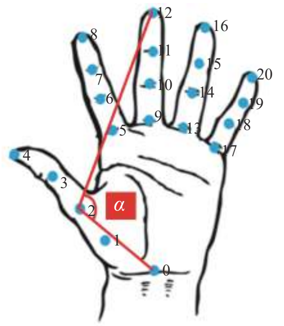

Fig. 6 Corresponding positions of 21 hand joints

### 3.2 Feature 2: The length

Using the coordinate of the joint points obtained by the two devices, the length information could be easily calculated. It could be found that the position of the fingertip is the most variable point, so we use (12) to calculate the following distances: 1) the distance between the root of the fingers and the fingertips, 2) the distance between the root of the palm (point 0 in Fig. 6) and each fingertip, 3) the distance between each fingertip.

$$length = \sqrt{(x_i - x_j)^2 + (y_i - y_j)^2} \tag{12}$$

where 1) $i$ = 2, 5, 9, 13, 17; $j$ = 4, 8, 12, 16, 20; 2) $i$ = 0; $j$ = 4, 8, 12, 16, 20; 3) $i$ = 4, 8, 12, 16, 20; $j$ = 4, 8, 12, 16, 20. (Note: $i$ and $j$ are not equal at the same time.) The algorithm flow is as follows.

**Algorithm 1.** Calculation of the length

**Input:** The coordinates of 21 hand joint points $(x_i, y_i)$, $(x_j, y_j)$.

**Output:** The length information.

1) **for** $i \in [0,2,4,5,8,9,12,13,16,17,20]$ **do**
2) **for** $j \in [4,8,12,16,20]$ **do**
3) **if** $i \neq$ j **then**
4) $length = \sqrt{(x_i - x_j)^2 + (y_i - y_j)^2}$
5) **end if**
6) **end for**
7) **end for**
8) **return** $length$;

### 3.3 Feature 3: The angle

Using the obtained joint point coordinates, we could easily calculate the angle information. We use (13)−(15) to calculate the following feature: 1) take the points 2, 5, 9, 13, 17 as the vertices, and the angle formed by the point 0 and any one of the points 4, 8, 12, 16, and 20, an example is shown in Fig. 6; 2) an angle formed by any three points in the fingertips.

$$\boldsymbol{A}_1 = (x_{i1} - x_j, y_{i1} - y_j) \tag{13}$$

$$\boldsymbol{A}_2 = (x_{i2} - x_j, y_{i2} - y_j) \tag{14}$$

$$\alpha = \arccos\left(\frac{\boldsymbol{A}_1 \cdot \boldsymbol{A}_2}{\|\boldsymbol{A}_1\|\|\boldsymbol{A}_2\|}\right) \tag{15}$$

where 1) $i1$ = 0; $i2$ = 4, 8, 12, 16, 20; $j$ = 2, 5, 9, 13, 17; 2) $i1$ = 4, 8, 12, 16, 20; $i2$ = 4, 8, 12, 16, 20; $j$ = 4, 8, 12, 16, 20. (Note: $i1$, $i2$, $j$ are not equal at the same time.) The algorithm flow is as follows.

**Algorithm 2.** Calculation of the angel

**Input:** The coordinates of 21 hand joint points $(x_{i1}, y_{i1})$, $(x_{i2}, y_{i2})$, $(x_j, y_j)$.

**Output:** The angel information.

1) **for** $j \in [2,5,9,13,17]$ **do**
2) **for** $i2 \in [4,8,12,16,20]$ **do**
3) $i1 = 0$
4) $\boldsymbol{A}_1 = (x_{i1} - x_j, y_{i1} - y_j)$
5) $\boldsymbol{A}_2 = (x_{i2} - x_j, y_{i2} - y_j)$
6) $\alpha 1 = \arccos\left(\frac{\boldsymbol{A}_1 \cdot \boldsymbol{A}_2}{\|\boldsymbol{A}_1\|\|\boldsymbol{A}_2\|}\right)$
7) **end for**
8) **end for**
9) **for** $i1, i2, j \in [4,8,12,16,20]$ **do**
10) **if** $i1 \neq i2 \neq j$ **then**
11) $\boldsymbol{A}_1 = (x_{i1} - x_j, y_{i1} - y_j)$
12) $\boldsymbol{A}_2 = (x_{i2} - x_j, y_{i2} - y_j)$
13) $\alpha 2 = \arccos\left(\frac{\boldsymbol{A}_1 \cdot \boldsymbol{A}_2}{\|\boldsymbol{A}_1\|\|\boldsymbol{A}_2\|}\right)$
14) **end if**
15) **end for**
16) **return** $\alpha 1, \alpha 2$

### 3.4 Feature 4: The new coordinates

In order to weaken the influence of different coordinate systems on the joint point coordinates, the coordinate origin could be unified as the root of the middle finger (point 9 in Fig. 6). Take the direction from point 0 to point 9 as the positive direction of the $y$-axis, the direction perpendicular to the $y$-axis and to the right is the positive direction of the $x$-axis.

The positive $y$-axis direction vector is expressed as

$$\boldsymbol{A}_y = (x_9 - x_0, y_9 - y_0). \tag{16}$$

The positive $x$-axis direction vector is expressed as

$$\boldsymbol{A}_x = \left(1, \frac{x_0 - x_9}{y_9 - y_0}\right). \tag{17}$$

The point representation in the new coordinate system is

$$length = \sqrt{(x_i - x_9)^2 + (y_i - y_9)^2} \tag{18}$$

$$\boldsymbol{A}_i = (x_i - x_9, y_i - y_9) \tag{19}$$

$$x_{inew} = length \times \frac{\boldsymbol{A}_i \cdot \boldsymbol{A}_x}{\|\boldsymbol{A}_i\|\|\boldsymbol{A}_x\|} \tag{20}$$

$$y_{inew} = length \times \frac{\boldsymbol{A}_i \cdot \boldsymbol{A}_y}{\|\boldsymbol{A}_i\|\|\boldsymbol{A}_y\|} \tag{21}$$

where $i = 0, 1, 2, \cdots, 20$. Through the above formulas, 21 point coordinates could be converted to a new coordinate system. We convert the original coordinates of the RGB Camera and Leap Motion to obtain the new coordinates of the 21 joint points. The algorithm flow is as follows.

**Algorithm 3.** Calculation of the new coordinates

**Input:** The coordinates of 21 hand joint points $(x_{i1}, y_{i1})$, $(x_{i2}, y_{i2})$, $(x_j, y_j)$.

**Output:** The new coordinates information.

1) $\boldsymbol{A}_y \leftarrow (x_9 - x_0, y_9 - y_0)$
2) $\boldsymbol{A}_x \leftarrow \left(1, \dfrac{x_0 - x_9}{y_9 - y_0}\right)$
3) **for** $i \in [0, 1, 2, \cdots, 20]$ **do**
4) $length = \sqrt{(x_i - x_9)^2 + (y_i - y_9)^2}$
5) $\boldsymbol{A}_i = (x_i - x_9, y_i - y_9)$
6) $x_{inew} = length \times \dfrac{\boldsymbol{A}_i \cdot \boldsymbol{A}_x}{\|\boldsymbol{A}_i\|\|\boldsymbol{A}_x\|}$
7) $y_{inew} = length \times \dfrac{\boldsymbol{A}_i \cdot \boldsymbol{A}_y}{\|\boldsymbol{A}_i\|\|\boldsymbol{A}_y\|}$
8) **end for**
9) **return** $x_{inew}, y_{inew}$

## 4 Experiment

### 4.1 Experimental setup

This section mainly introduces the relevant parameter settings of the algorithms used in the experiment. The relevant parameter settings of support vector machine (SVM) and BLS are shown in Table 2.

We use a BP neural network in this paper, and the number of nodes in the input layer is determined by the dimension $N$ of each feature described in Section 3. The number of nodes in the first and second layers is set as $0.5N - 2N$, and the number of nodes in the output layer is set as 10. Fig. 7 shows the transfer process between different devices.

### 4.2 Experimental results

In Section 3, the different gesture features of the RGB Camera and Leap Motion are obtained. This section will use the algorithm introduced in Section 2 to transfer the data of these two domains.

#### 4.2.1 Experiment 1: Transfer of raw data collected by two devices

The results of Experiment 1 are shown in Fig. 8. It could be found from the experimental results that if the images taken by the RGB Camera and the coordinates of joint points obtained by the Leap Motion are transferred to each other, the experimental results are poor.

#### 4.2.2 Experiment 2: Mutual transfer of coordinate features

The results of Experiment 2 are shown in Fig. 9. From the experimental results of Experiment 2, the following conclusions could be drawn:

1) By comparing with Experiment 1, we could see that after extracting the coordinate features, the accuracy of the transfer result between the two devices has been greatly improved.

2) The JDA algorithm could reduce the distance between two domains, and improve the accuracy of the experimental results in most cases.

#### 4.2.3 Experiment 3: Mutual transfer of length features

The results of Experiment 3 are shown in Fig. 10.

#### 4.2.4 Experiment 4: Mutual transfer of angle features

The results of Experiment 4 are shown in Fig. 11.

Table 2 SVM and BLS related parameter settings

| Related parameter | Ranges |
|---|---|
| $C$ | 1, 2, ⋯, 20 |
| $\gamma$ | 0.000 1, 0.001, ⋯ , 1 |
| $p$ | 5, 10, ⋯, 30 |
| $n$ | 5, 10, ⋯, 30 |
| $q$ | 20, 40, ⋯ , 300 |

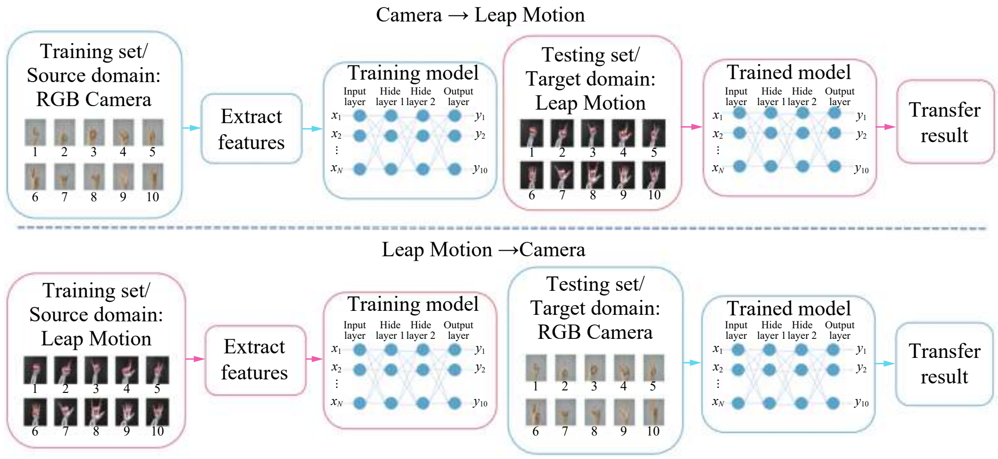

Fig. 7 Process of transfer between different devices

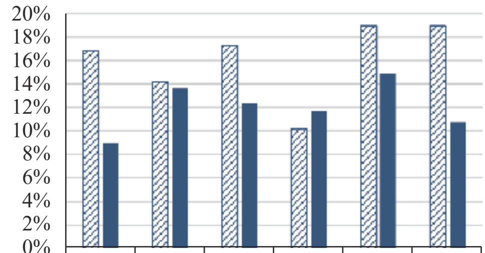

| | SVM | BLS | NN | JDA+SVM | JDA+BLS | JDA+NN |
|---|---|---|---|---|---|---|
| Camera → Leap Motion | 16.87% | 14.19% | 17.31% | 10.18% | 19.02% | 18.96% |
| Leap Motion →Camera | 9.03% | 13.69% | 12.44% | 11.76% | 14.89% | 10.80% |

Fig. 8 Comparison of results in Experiment 1

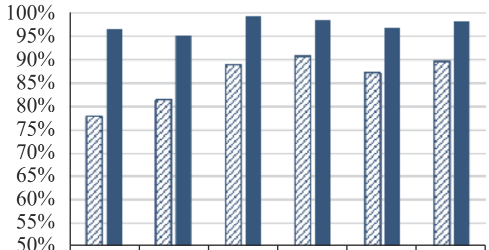

| | SVM | BLS | NN | JDA+SVM | JDA+BLS | JDA+NN |
|---|---|---|---|---|---|---|
| Camera → Leap Motion | 77.84% | 81.39% | 88.90% | 90.76% | 87.22% | 89.67% |
| Leap Motion →Camera | 96.54% | 95.32% | 99.32% | 98.65% | 96.85% | 98.29% |

Fig. 9 Comparison of results in Experiment 2

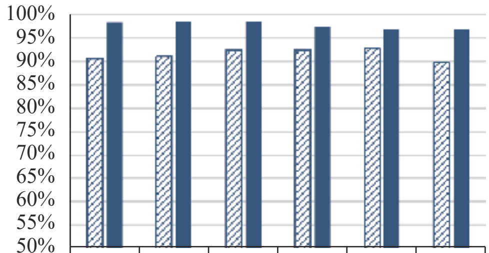

| | SVM | BLS | NN | JDA+SVM | JDA+BLS | JDA+NN |
|---|---|---|---|---|---|---|
| Camera → Leap Motion | 90.60% | 91.17% | 92.51% | 92.51% | 92.73% | 89.75% |
| Leap Motion →Camera | 98.42% | 98.65% | 98.55% | 97.45% | 96.95% | 96.97% |

Fig. 10 Comparison of results in Experiment 3

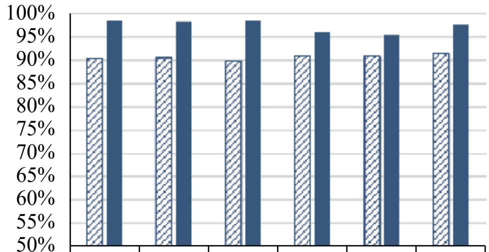

| | SVM | BLS | NN | JDA+SVM | JDA+BLS | JDA+NN |
|---|---|---|---|---|---|---|
| Camera → Leap Motion | 90.46% | 90.54% | 89.89% | 91.04% | 90.97% | 91.45% |
| Leap Motion →Camera | 98.56% | 98.40% | 98.54% | 96.24% | 95.49% | 97.62% |

Fig. 11 Comparison of results in Experiment 4

##### 4.2.5 Experiment 5: Transfer between two devices after coordinate conversion

The results of Experiment 5 are shown in Fig. 12.

From the comparison of the experimental results of Experiments 3–5, it could be found that most of the experimental results are improved little or even not at all by using the JDA algorithm. Through the analysis, we could see that because of the length feature, the angle feature and the new coordinate feature are less affected by the original coordinate system. Different original coordinate systems do not have much influence on them. For this paper, the main function of the JDA algorithm is to reduce the impact of different coordinate systems on the data, thus reducing the difference between domains. According to [31], we know that the JDA algorithm needs complex calculations to obtain the transformation matrix, which is a time-consuming process. In this paper, we could directly use the extracted length, angle and coordinate features to transfer learning, which not only guarantees the accuracy, but also greatly reduces the training time.

By comparing the 5 experiments, it could be found that the average accuracy of experiment 5 is the highest. In other words, the best results are obtained by the new coordinates feature. In addition, in five experiments, the accuracy of the Leap Motion transfer to the RGB Camera is generally higher than that of the RGB Camera transfer to the Leap Motion. After analysis, we think that this is because the coordinates originally extracted by Leap Motion are in three-dimensional space, while those extracted from the RGB Camera images are two-dimensional coordinates. Therefore, the features of Leap Motion have more information, the accuracy of transfer is higher when Leap Motion data is used as the source domain.

### 5 Discussions

Some interesting phenomena are found when using the neural network algorithm to transfer and classify data. Figs. 13(a)–13(c) show the experimental results with the Camera as the source domain and the Leap Motion as the

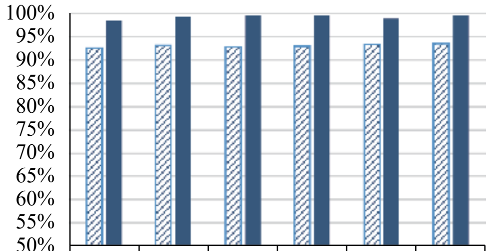

| | SVM | BLS | NN | JDA+SVM | JDA+BLS | JDA+NN |
|---|---|---|---|---|---|---|
| Camera → Leap Motion | 92.59% | 93.25% | 92.83% | 93.08% | 93.44% | 93.52% |
| Leap Motion →Camera | 98.56% | 99.37% | 99.66% | 99.66% | 98.93% | 99.65% |

Fig. 12 Comparison of results in Experiment 5

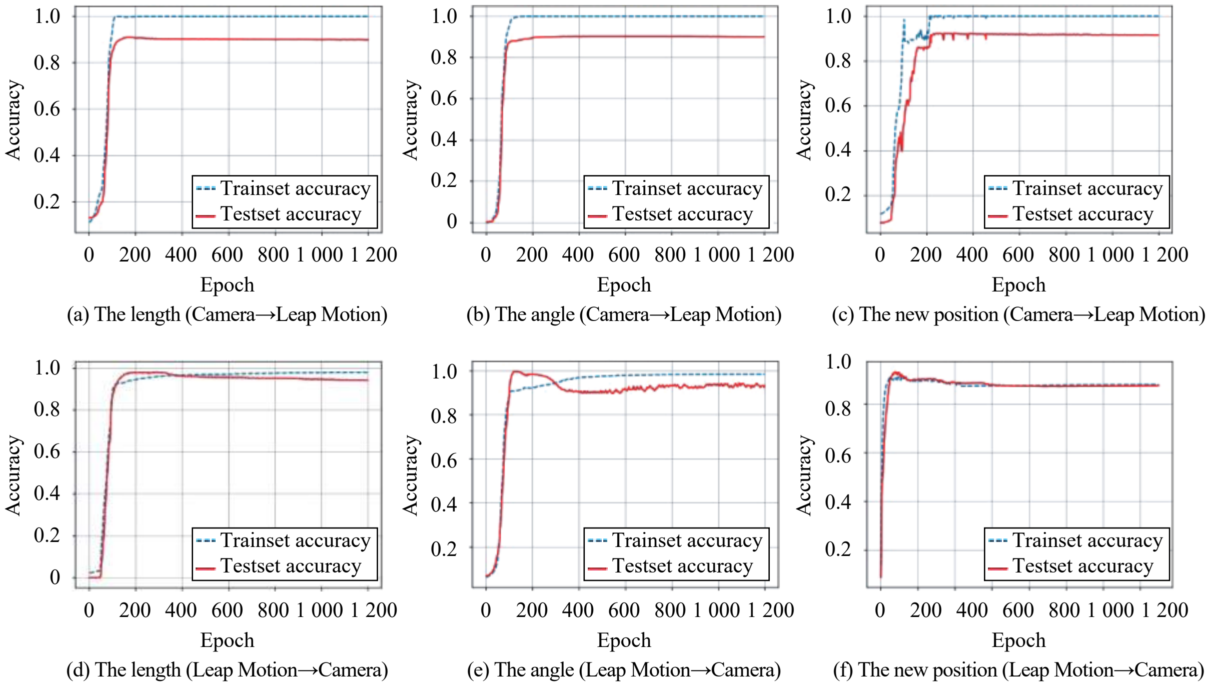

Fig. 13 Comparison of experimental results using neural network classification target domain. Figs. 13(d)−13(f) show the experimental results with the Leap Motion as the source domain and the Camera as the target domain. It could be seen from the comparison figure that when the Camera (training set/source domain) transfers to the Leap Motion (test set/target domain), the accuracy of the target domain is generally lower than the source domain. But something special happened when the Leap Motion (training set/source domain) transfers to the Camera (test set/ target domain), which will be discussed in more detail below.

From the experimental results shown in Figs. 13(d)−13(f), we could draw the following conclusions:

1) In a small interval to the left of the intersection (the rightmost intersection), the accuracy of the target domain is higher than the source domain, and the highest point of the accuracy of the target domain is in this interval. This means that in this interval, the model trained on the source domain is more suitable for the target domain. We speculate that this is because the Leap Motion data is originally in three-dimensional space, while the Camera data is in two-dimensional space. In other words, the Leap Motion has a more abundant feature space than the Camera, so that the Camera data could perform better. Therefore, in this interval, the accuracy of the target domain is higher than the source domain.

2) In the right region of the intersection (the rightmost intersection), the accuracy of the target domain decreases with the improvement of source domain accuracy. This may mean that the model is more suitable for the source domain due to the increase of training times, which reduces the generalization ability in the target domain. Therefore, it could be concluded that in some cases, the training times in the source domain affect the accuracy of the target domain.

For transfer learning, we have not yet found the discussion of these two points. Compared with the discussion in [33] about "1) Which layers in the source domain could be transferred to the target domain? 2) How much layers of knowledge in the source domain are transferred to the target domain?" We propose "When is the best time to transfer during the training of the source domain." A detailed introduction is given based on the experiment.

### 6 Conclusions

In this paper, an effective transfer learning method for gesture recognition between the RGB Camera and Leap Motion has been put forward. The different distribution of data collected by the Leap Motion and the RGB Camera raises challenging problems, for which effective solutions have been presented. We extracted various features from the obtained original data, such as the coordinates, the length and the angle features, and compared the learning performances with and without using the JDA algorithm. The experimental results show the performance of different features when using different algorithms. Through the comparison of several groups of experimental results, we found that the average accuracy of the new coordinate features is the highest. In the future work, we will focus on the following points:

1) At present, only two-dimensional features are used in the transfer learning of gesture recognition, which has certain limitations on the direction of the palm. If the palm is not parallel to the device, it will have an impact on the classification results. We will use Kinect to extract more reliable features from 3D space.

2) We only discuss the experiment result of coordinates, length, and angle features, more features could be calculated for transfer.

3) In the future, it could also be extended to the field of transfer learning of the action recognition among different devices.

### Acknowledgements

This work was supported by National Nature Science Foundation of China (NSFC) (Nos. U20A20200, 61811530281, and 61861136009), Guangdong Regional Joint Foundation (No. 2019B1515120076), Fundamental Research for the Central Universities, and in part by the Foshan Science and Technology Innovation Team Special Project (No. 2018IT100322).

### Open Access

This article is licensed under a Creative Commons Attribution 4.0 International License, which permits use, sharing, adaptation, distribution and reproduction in any medium or format, as long as you give appropriate credit to the original author(s) and the source, provide a link to the Creative Commons licence, and indicate if changes were made.

The images or other third party material in this article are included in the article's Creative Commons licence, unless indicated otherwise in a credit line to the material. If material is not included in the article's Creative Commons licence and your intended use is not permitted by statutory regulation or exceeds the permitted use, you will need to obtain permission directly from the copyright holder.

To view a copy of this licence, visit http://creativecommons.org/licenses/by/4.0/.
